# Imagining Others’ Minds: The Positive Relation Between Children’s Role Play and Anthropomorphism

**DOI:** 10.3389/fpsyg.2018.02140

**Published:** 2018-11-13

**Authors:** Rachel L. Severson, Shailee R. Woodard

**Affiliations:** Department of Psychology, University of Montana, Missoula, MT, United States

**Keywords:** anthropomorphism, pretense, role play, imagination, children, simulation theory

## Abstract

Children’s role playing, whether personifying toys or imagining invisible friends, involves imagining others’ minds and internal states. Similarly, anthropomorphism – the attribution of internal states to non-human others (e.g., animals, inanimate nature, or technologies) – also involves imagining others’ minds and internal states. We propose that the imaginative process of simulating and projecting internal states is common to both role play and anthropomorphism. The current study investigated the relation between children’s role play and anthropomorphism. Ninety children (5, 7, and 9 years) were administered Individual Differences in Anthropomorphism Questionnaire – Child Form (IDAQ-CF), comprised of the technology-inanimate nature and animal subscales, and the Role Play Scale, which assessed (a) impersonation of animals, people, and/or machines and (b) imaginary companions (ICs), including invisible friends and personified toys. Results indicated that the imaginative act of impersonating an animal, person, and/or machine was positively related to anthropomorphism, and specifically anthropomorphism of inanimate nature and technology. Second, anthropomorphism of animals was highest amongst children with invisible ICs, followed by those with toy ICs and those who impersonated. Finally, children who frequently engaged with an invisible ICs more readily anthropomorphized in general and technology and inanimate nature in particular relative to all other children. Results are discussed in terms of the differing degrees of imagination involved in anthropomorphism of animals versus technology and inanimate nature.

## Introduction

“…if she brought home a flower, or a pebble she always brought several flowers or pebbles at the same time so they should have company and not feel lonely” (Piaget, 1929, p. 209).

Young children often endow inanimate objects with a range of internal states (e.g., emotions, thought, and desires) and these attributions, as the above quote illustrates, can guide children’s behavior. Piaget’s (1929) seminal work on animism – children’s tendency to attribute consciousness and life to inanimate objects – provided detailed observations and a theoretical framework to explain this tendency and, in turn, inspired decades of developmental research to uncover the nature of children’s conceptions (e.g., Gelman and Spelke, 1981; Carey, 1985; Gelman, 2003; Inagaki and Hatano, 2006). This corpus of work has shown that, contrary to Piaget’s assertion, young children (by age 3) are quite capable of distinguishing between animates and inanimates in terms of movement (Gelman and Gottfried, 1996), biology (Gelman, 2003), and psychological properties (Gelman and Spelke, 1981). And yet this explanation seemingly fails to account for children’s widespread tendency to attribute human-like mental states to inanimate entities, what is often referred to as anthropomorphism (Waytz et al., 2010a; Severson and Lemm, 2016). That is, if young children distinguish between animates and inanimates (for example, understanding that rocks are inanimate while dogs are animate), how do we understand the numerous instances in which this distinction appears to be blurred, such as when the child brings home several rocks or flowers so that none are lonely?

One possibility is that children are pretending. Consider, for example, children’s propensity to personify toys and stuffed animals. It seems reasonable that children may not be sincere in their attributions of internal states and personalities to such artifacts. In fact, Gelman et al. (1983) suggested that children were rarely animistic *except* when they were induced to answer in “play mode.” Yet, is it the case that children attribute internal states to inanimate entities *only* in the context of pretense? Or might their attributions reflect their veridical beliefs? Piaget (1929) viewed animism in the context of play as a separate endeavor (and, indeed, deferred discussion of it in his treatise on animism, p. 207). Moreover, there is evidence that children can be quite sincere (i.e., not in play mode) when attributing animate characteristics to objects (e.g., Kahn et al., 2006), and in particular when they are attributing *psychological* characteristics (Waytz et al., 2010a; Severson and Lemm, 2016). In short, children ascribe internal states to objects in the context of pretense, but they also make those assertions quite seriously (i.e., anthropomorphism). Thus, it is important to understand anthropomorphism as a pervasive phenomenon that goes beyond mere pretense.

Although we argue that pretense and anthropomorphism are distinct, they nevertheless appear to involve conceptually related processes. Children’s anthropomorphism – the attribution of internal states to non-human others (e.g., animals, inanimate nature, or technologies) – involves imagining others’ minds and internal states. Similarly, pretense, whether personifying toys or imagining invisible friends (i.e., role play), also involves imagining others’ minds and internal states. Thus, it may be that the process of imagining others’ internal states is common to both anthropomorphism and pretend role play. The current study seeks to investigate the relation between anthropomorphism and pretend role play in children 5, 7, and 9 years as a starting point toward understanding whether both might draw upon a common imaginative process. Although this study does not directly assess the underlying processes in either anthropomorphism or role play, it represents an initial step in establishing whether there is a pattern of association between these phenomena.

### Role Play

Pretend play is a hallmark of childhood. Children pretend to be a favorite character or fierce animal, they endow stuffed animals with elaborate personalities, and even create entirely imagined companions that can have an appreciable presence despite being invisible (e.g., a place setting at the table; Taylor and Carlson, 2002). Collectively referred to as ‘role play,’ these forms of pretense include impersonation of other people, animals, or machines, as well as creation of imaginary companions (ICs), whether a stuffed animal, toy, or an invisible friend (Harris, 2000). In this way, role play is distinct from solitary or joint pretend play involving object substitution (e.g., substituting a banana for a phone) (Taylor and Carlson, 1997; Harris, 2000).

According to Simulation Theory, role play is thought to involve a dual process of simulation and projection (Harris, 2000). That is, children imagine (or simulate) internal states (e.g., perspectives, emotions, thoughts) and project those internal states onto either themselves (in the case of impersonation) or an IC, whether a stuffed animal or an invisible friend. Further, Harris (2000) argues that role play and theory of mind are conceptually related, as both involve simulation of mental states, and only the target of the simulation differs (e.g., an imaginary friend in the case of role play and a person in the case of theory of mind). Perhaps not surprisingly then, children who have invisible ICs tend to perform better on standard measures of theory of mind (Taylor and Carlson, 1997) and have better mental representation abilities (Taylor et al., 1993). In other words, imagining others’ minds – whether pretend others (role play) or human others (theory of mind) – is positively related.

Role play is quite prevalent in childhood. Nearly all children (95–100%) engage in impersonation and do so at the same rate from preschool (3–4 years) to early school age (6–7 years) (Taylor and Carlson, 1997; Taylor et al., 2004), with boys showing higher rates of impersonation (Carlson and Taylor, 2005). Further, roughly two-thirds of children (age 3–7 years) have an IC (toy or invisible) although the type of ICs children create changes with age (Taylor et al., 2004). Taylor et al. (2004) found that preschoolers were equally divided between invisible friends (48%) and personified toys (52%), whereas 6- and 7-year-olds were more likely (67%) to have invisible friends than personified toys (33%). The prevalence of ICs declines markedly by age 9 and beyond, with approximately one-third of 9-year-olds and only 9% of 12-year-olds reporting having an invisible IC (Pearson et al., 2001). Moreover, children vary in the frequency with which they engage in the different forms of role play. For example, Carlson and Taylor (2005) found that roughly half of the 3- and 4-year-olds in their study reported having an invisible companion and the majority reported impersonating. However, when considering the frequency with which children engaged in these forms of role play (based on parent-report), the prevalence reduced considerably when limited to frequent pretenders (28% for those with ICs, 19% for impersonators). Thus, children’s level of engagement in terms of frequency has been an important criterion to distinguish children who are high versus low in role play (e.g., Taylor and Carlson, 1997; Carlson and Taylor, 2005). And this distinction is meaningful. For example, the positive association with theory of mind was found in those children who frequently engaged in role play (Taylor and Carlson, 1997).

It is also the case that the forms of role play are uniquely related to individual differences in social cognitive and imaginative abilities. As mentioned previously, preschool children with invisible ICs have more advanced theory of mind and mental representation abilities (Taylor et al., 1993; Taylor and Carlson, 1997). Children with invisible ICs may have greater imaginative abilities due in part to the fact that their ICs are completely imaginary, rather than relying upon a physical toy that often provides some suggestions of persona or a character or persona that a child embodies. Indeed, relative to their counterparts who endow stuffed animals or toys with elaborate personalities, children with invisible ICs have advanced visual imagery abilities (Tahiroglu et al., 2011). On the other hand, 6- and 7-year-olds who readily impersonated other people and characters (compared to those who do not) demonstrated better emotional understanding, yet 6- and 7-year-olds with ICs showed no advantage in emotional understanding (despite the previous relation as preschoolers) (Taylor et al., 2004). In short, although the forms of role play are theorized to involve a common process of mental simulation, there is evidence that the role play types may be differentially associated with certain cognitive abilities. As a result, and as others have argued (e.g., Harris, 2000; Carlson and Taylor, 2005), the forms of role play should be considered separately.

### Anthropomorphism

Anthropomorphism also involves imagining others’ minds. At its core, anthropomorphism refers to the attribution of humanlike minds and internal states to non-humans (Epley et al., 2007; Severson and Lemm, 2016), although some also conceptualize anthropomorphism as including attributions of humanlike physical features (e.g., Guthrie, 1993; Barrett and Richert, 2003; see Waytz et al., 2010a for an overview). In the act of anthropomorphizing, people may ascribe humanlike emotions, beliefs, desires, knowledge, intentions, sociality, and moral worth and responsibility to non-human entities (Epley et al., 2007; Severson and Carlson, 2010). Importantly, these attributions are independent of biology – that is, children attribute psychological states to technologies while simultaneously judging them as non-biological (e.g., Kahn et al., 2006, 2012; Jipson and Gelman, 2007; Melson et al., 2009) – suggesting that anthropomorphism is related to mentalizing rather than biological concepts.

Not only are the features one may attribute when anthropomorphizing quite broad, the targets are also widely varied. Humans anthropomorphize animals, inanimate nature, natural phenomena, supernatural entities, illnesses, objects, and technologies (e.g., Chin et al., 2005; Waytz et al., 2010a; Shahar and Lerman, 2012). Additionally, Guthrie (1993) theorizes that anthropomorphism is a universal human tendency (see also Zawieska et al., 2012). This notion is substantiated by the high prevalence of anthropomorphism among children and adults (Bloom, 2007; Waytz et al., 2010a; Severson and Lemm, 2016), and the fact that it is so far-reaching, both in terms of the subject matter that is anthropomorphized and in the variety of peoples that anthropomorphize (Epley et al., 2007).

Although anthropomorphism is often conceptualized as a unified construct, it is important to note that critical distinctions exists depending upon the class of non-human entities. First, anthropomorphism of animals, technology, and inanimate nature is independent from the anthropomorphism of supernatural (or spiritual) entities (Waytz et al., 2010a, Study 1; Willard and Norenzayan, 2013). Second, anthropomorphism of animals is distinct from anthropomorphism of inanimate nature and technology (although they are correlated), and both children and adults anthropomorphize animals to a greater degree than inanimate nature and technology (Waytz et al., 2010a, Study 2; Severson and Lemm, 2016; Li et al., 2017).

Why then do people anthropomorphize? Several non-mutually exclusive explanations have been put forth to explain this common human tendency. Broadly speaking, anthropomorphism may result from internal (human) motivations, overextension of cognitive mechanisms, or external (entity) factors. First, individuals anthropomorphize in order to fill in gaps in their knowledge of non-human entities. Indeed, individuals are more likely to anthropomorphize qualities of non-human entities that are not readily observed, such as internal states (Epley et al., 2007). Barrett and Richert (2003) likewise suggest that, when necessary, people anthropomorphize in order to “fill in the blanks” in their cognition. For example, because it is not possible to fully comprehend the experiences of our pets, people default to what they know best – their own emotional experience. That is, individuals often extend to their pet the same complex human emotions a person would experience when they are left alone for a long period of time or separated from their birth families as puppies. The anthropomorphism that occurs in these situations is likely due to the basic human motivation to understand one’s environment and possess some degree of agency over it. Anthropomorphism fulfills these basic human desires by making non-human entities appear similar to oneself and thus reduces the “uncertainty, unpredictability, and randomness” that results from a sense of low agency (Waytz et al., 2010b, p. 424).

Second, it is also possible that anthropomorphism results from an overextension of one’s social cognition (e.g., Boyer, 2001). In typical circumstances, adults and children utilize their theory of mind to conceptualize and make predictions regarding other individuals’ internal states. Yet, children (and adults) may apply their reasoning about others’ minds more broadly. That is, children may use their theory of mind to seek to understand non-human others’ actions and internal states. Indeed, during the preschool period in which theory of mind development is most marked, children more readily anthropomorphize non-human entities (Tahiroglu, 2012). It stands to reason that children encounter difficulties in determining which entities have internal states and how human-like their internal states may be. In this way, the overextension of social cognition is related to filling in the gaps in one’s knowledge (discussed above). What follows is that children overgeneralize their developing theory of mind and endow animals and other non-human entities with internal states similar to their own. In fact, research suggests that infants attribute minds to anything that exhibits self-propelled movement and behavior that follows a stimulus from the environment (e.g., Gergely et al., 1995; Csibra et al., 1999).

Third, external factors particular to the entity being anthropomorphized may contribute to one’s tendency to anthropomorphize. For example, certain entities provide external or behavioral cues that are suggestive of internal states. Waytz et al. (2013) have termed these ‘target triggers.’ Animals display various behaviors that are readily interpreted as indicative of their emotions. A wagging tail indicates happiness. A nip indicates anger or annoyance. Even the addition of eyes to simple shapes (e.g., circles or spheres) is enough to suggest internal states to infants (e.g., Johnson et al., 1998; Hamlin et al., 2007). Entities with robust or numerous external mental states cues are more readily anthropomorphized than those with comparatively weak or few target triggers, as evidenced by the higher rates of anthropomorphism of animals compared to technology and inanimate nature (e.g., Waytz et al., 2010a; Severson and Lemm, 2016). Indeed, it may be that entities that lack external cues of agency and mental states require more motivation or imagination on the part of the individual to view such entities in anthropomorphic terms. Thus, the tendency to anthropomorphize may be due to factors within an individual (e.g., filling in the gaps in their knowledge or overextension of social cognition) or to features or behaviors of the entity that is the target of anthropomorphism (e.g., eyes or a wagging tail), or a combination of both.

Although anthropomorphic beliefs are relatively stable in adulthood (Waytz et al., 2010a), there is evidence that anthropomorphic beliefs undergo developmental changes. However, the early evidence is somewhat mixed in terms of the specific trajectory. Some studies have found age-related increases in anthropomorphism. For example, 5-year-olds and adults were more likely than 3- and 4-year-olds to perceive internal states in Heider and Simmel’s (1944) movie of animated shapes (Springer et al., 1996). In other work, 4- and 6-year-olds did not differ in anthropomorphism of animals (Li et al., 2017), yet 9-year-olds were more likely than 5-year-olds to anthropomorphize animals (Severson and Lemm, 2016). Taking these studies together, 5-year-olds appear quite adult-like when using movement as a cue to infer internal states to simple geometric shapes, and further age-related changes are evident between 5 and 9 years when endorsing anthropomorphic beliefs about animals. Still, other studies show no significant age-related changes in anthropomorphism of technology and inanimate nature between 4- and 6-year-olds (Li et al., 2017) or 5-, 7-, and 9-year-olds (Severson and Lemm, 2016), although descriptively younger children endorsed more anthropomorphic beliefs about technology and inanimate nature. The effect sizes in these studies were small to medium (Cohen’s *d* ranged from 0.29 to 0.55) suggesting they may have been underpowered to detect these effects (although note that age-related effects were not the primary goal of either study). Thus, the research suggests there are differing developmental trajectories for anthropomorphism of animals versus inanimate nature and technology.

### Theoretical Implications of a Relation Between Role Play and Anthropomorphism

Why might a relation between role play and anthropomorphism be of interest? We suggest there are important theoretical implications for such a relation. According to Simulation Theory (Harris, 2000), the imaginative process of simulating and projecting internal states is theorized to be involved in both role play and social cognition. Building on this idea, we further suggest that anthropomorphism may draw upon the same imaginative process. Indeed, it may be the case that the simulation process is common to mentalizing more generally, whether imagining the internal states of another person (social cognition), a non-human entity (anthropomorphism), or an imaginary friend (role play). Although the underlying cognitive process may be similar, it follows that separate additional processes would also be involved, for example, the self-other distinction in social cognition, the fantasy-reality distinction in role play, and the animate–inanimate distinction in anthropomorphism.

In line with this reasoning, several studies suggest a relation between social cognition, role play, and anthropomorphism. Tahiroglu (2012) found a positive relation between role play and anthropomorphism in both adults and children (4–6 years). Moreover, Castelli et al. (2000, 2002) found neural activation of the ‘mentalizing’ network in response to anthropomorphized animated shapes, although this pattern of activation was not evident among adults with autism (Castelli et al., 2002). Yet, research using other measures have produced mixed results. Tahiroglu (2012) found evidence in 4- to 6-year-olds of a relation between false belief understanding (false contents task) and anthropomorphism (using an interview-style measure), but this relation was not significant using other theory of mind measures and a narrative measure of anthropomorphism of animated shapes (akin to the procedure used by Castelli et al., 2000). Recent work suggests that adults with autism personify objects at higher rates than non-autistic adults (White and Remington, 2018) – a result that is striking given the typical deficits in theory of mind among individuals with autism (see also Atherton and Cross, 2018).

The mixed results point to the need for further investigation into the potential relation between social cognition, role play, and anthropomorphism. Answers to these questions could have important implications for our understanding of each of these constructs, individually as well as how they may relate to each other. It is therefore of theoretical interest to explore the bounds of simulating others’ minds (i.e., whether this imaginative process also explains anthropomorphism). If so, it will be important to understand the nature of the relation (e.g., causally related or based on a common underlying mechanism), how other cognitive processes may uniquely operate within each context (social cognition, role play, and anthropomorphism), and whether any relation holds in atypical populations (e.g., individuals on the autism spectrum).

### The Current Study

Thus, the purpose of the current study was to examine the relation between individual differences in role play and anthropomorphism in children 5, 7, and 9 years old. We focused on this age range as anthropomorphism and role play are prevalent during this period, although with slightly different trajectories and timeframes. Role play is equally prevalent in children from 3 to 7 years and declines by age 9 (Taylor et al., 2004), whereas anthropomorphism (of animals) increases from age 5 to 9 (Severson and Lemm, 2016). Thus, although we had no *a priori* predictions of age-related changes in the relation between role play and anthropomorphism, we sought to assess the relation across a broader age range in order to capture potentially important developmental shifts, particularly as role play decreases and anthropomorphism increases. Importantly, if a positive relation between role play and anthropomorphism exists independent of age, it is reasonable to further consider how they are related and whether they rely upon a common underlying process. Thus, as an initial step in examining the relation between these constructs, it is important to consider whether the relation is temporally bound to a particular age or if it holds across age groups.

Two main questions structured our investigation. First, is there a positive relation between children’s engagement in role play and anthropomorphism? To our knowledge, only one previous study (Tahiroglu, 2012) found correlational evidence of such a link in 4- to 6-year-olds, thus we sought to replicate the earlier finding with a broader (and older) age range. Second, we reasoned that higher levels of imagination in role play (i.e., children with invisible ICs and/or high frequency of role play) would be related to forms of anthropomorphism that involve greater imagination (i.e., attributing internal states to inanimate nature and technology). In other words, we posited that the specific link between role play and anthropomorphism is based on individual differences in one’s tendency to engage in the simulation process to imagine others’ mental states. These individual differences may result from differences in simulation ability, wherein some children are more facile in the simulation process and readily do so across domains (pretense and anthropomorphism). Or individual differences may result from differences in children’s experience simulating mental states, such that repetitively engaging in simulation in one domain (pretense) may lead to more simulation in another domain (anthropomorphism, or vice versa). Accordingly, individuals with greater imaginative (i.e., simulation) abilities in role play should show a corresponding proclivity to anthropomorphize, and especially so with entities that provide few to no cues of internal states (e.g., inanimate nature), and thereby placing higher demands on the child’s ability to imagine. Thus, the second question asked whether more sophisticated forms of role play differentially relate to anthropomorphic tendencies?

## Materials and Methods

### Participants

Participants included 90 children ages 5 (*n* = 30; *M_age_* = 5.5, *SD* = 0.28; 50% girls), 7 (*n* = 30; *M_age_* = 7.4, *SD* = 0.32; 50% girls), and 9 (*n* = 30; *M_age_* = 9.4, *SD* = 0.24; 50% girls) years. The majority of participants were White (73%), with the remaining participants indicating their race/ethnicity as more than one race/ethnicity (18%), Latino/a (3%), Asian (2%), and Other (9%). Participants were recruited through flyers distributed throughout the community and announcements in school newsletters. This study was carried out in accordance with the recommendations of Human Subjects Division of the WWU Research Compliance Office, Institutional Review Board (IRB) Committee. The protocol was approved by the IRB Committee. All parents of participating children gave written informed consent in accordance with the Declaration of Helsinki, and participating children provided assent. Each participant received a t-shirt and $5 for their participation.

### Measures and Procedure

The study was conducted at a university research laboratory in Bellingham, WA, United States. Following the consent/assent process, participants were individually administered the Individual Differences in Anthropomorphism Questionnaire – Child Form (IDAQ-CF) followed by the Role Play Scale. The data for the current study come from a larger study on children’s conceptions of a social robot and a puppet, in which we investigated the factor structure and predictive validity of the IDAQ-CF in a child sample (Severson and Lemm, 2016, Study 2). The measures in the current study were the first two administered in the larger study’s procedure, and the subsequent measures focused on children’s conceptions of a social robot and puppet (e.g., familiarization phase, free play, attribution interview).

#### IDAQ-CF

The IDAQ-CF assesses individual differences in children’s anthropomorphism of technologies, inanimate nature, and animals (Severson and Lemm, 2016). The IDAQ-CF was adapted for use with children from the adult version of the IDAQ (Waytz et al., 2010a). Like the adult version, the IDAQ-CF consists of two correlated factors: One assessing anthropomorphic beliefs about technology and nature (Technology-Nature subscale) and the other assessing anthropomorphic beliefs about animals (Animal subscale). We refer the interested reader to Severson and Lemm (2016) for a detailed description of the development and validation of the IDAQ-CF.

Participants were first trained on a two-part question format. The first part consisted of a yes/no question (For example, “Do you like candy/broccoli/carrots?”) to which children responded using a ‘thumbs up’ (yes) or ‘thumbs down’ (no). ‘Yes’ responses were followed up with the second part of the question, “How much?” (For example, “How much do you like candy/broccoli/carrots?”), to which children were directed to answer on a scale with three increasingly tall bars labeled “a little bit,” “a medium amount,” and “a lot.” Thus, responses were coded on a 4-point scale: No (0), Yes-a little bit (1), Yes-medium amount (2), and Yes-a lot (3). The 12 IDAQ-CF test items were then presented in random order following this two-part question format (Table 1).

**Table 1:** Means (SD) on IDAQ-CF by age group.

IDAQ-CF items (presented in random order)		5 years *M* (*SD*)	7 years *M* (*SD*)	9 years *M* (*SD*)
Technology-Nature subscale	1. Does a car do things on purpose? [intention]	0.63 (1.10)	0.53 (1.01)	0.43 (0.86)
	2. Does a TV have feelings, like happy and sad? [emotion]	0.67 (1.06)	0.10 (0.55)	0.27 (0.74)
	3. Does a robot know what it is? [consciousness]	1.20 (1.22)	0.93 (1.14)	0.90 (1.19)
	4. Does computer think for itself? [mind]	0.97 (1.40)	0.43 (0.97)	1.07 (1.34)
	5. Does the wind do things on purpose? [intention]	1.23 (1.36)	0.59 (1.09)	0.60 (1.10)
	6. Does a mountain have feelings, like happy and sad? [emotion]	0.40 (0.86)	0.10 (0.40)	0.40 (0.93)
	7. Does the ocean know what it is? [consciousness]	0.47 (1.07)	0.33 (0.84)	0.37 (0.81)
	8. Does a tree think for itself? [mind]	0.53 (1.04)	0.40 (0.89)	0.57 (1.01)
	***Subscale Mean Score***	**0.76 (0.79)**	**0.44 (0.45)**	**0.61 (0.52)**
Animal subscale	9. Does a turtle do things on purpose? [intention]	0.77 (1.07)	1.41 (1.15)	1.79 (1.18)
	10. Does a cheetah have feelings, like happy and sad? [emotion]	2.13 (1.07)	1.93 (1.05)	2.27 (0.94)
	11. Does a lizard know what it is? [consciousness]	0.93 (1.26)	1.07 (1.03)	1.57 (1.20)
	12. Does an insect or bug think for itself? [mind]	1.20 (1.10)	1.53 (1.07)	1.90 (1.05)
	***Subscale Mean Score***	**1.26 (0.79)**	**1.49 (0.72)**	**1.90 (0.80)**
***Overall Mean Score***		**0.93 (0.64)**	**0.79 (0.37)**	**1.04 (0.43)**

#### Role Play Scale

We assessed children’s engagement in role play in terms of impersonation and ICs (adapted from Taylor and Carlson, 1997). The impersonation measure included child- and parent-report of the child’s impersonation of animals, other people (e.g., parent, doctor, teacher), and/or machines (e.g., car, airplane). That is, across three questions, children were asked if they had ever pretended to be an animal, another person, and/or a machine. Responses received a score of 1 for a “yes” response and 0 for a “no” response. Parents were also asked to report whether their child ever pretended to be an animal, a person, and/or a machine and, if so, the frequency in which the child engaged in this type of play (1 = rarely, 5 = frequently).

The IC measure similarly included child- and parent-report of ICs, including toys (e.g., stuffed animals) endowed with a stable personality and completely invisible ICs. Children who reported having an IC (now or in the past) were further interviewed in order to substantiate their claim. These questions probed details about the IC, including its name, age, gender, physical appearance, whether it was a toy or completely invisible (a forced-choice response), and characteristics the child liked or did not like about their IC. Children received a score of ‘1’ if they affirmed (and substantiated) that they had an IC and a score of ‘0’ if they denied having an IC (or affirmed having an IC but did not substantiate their claim). In addition, we categorized children’s ICs as ‘toy IC’ or ‘invisible IC’ based on their response to the forced-choice question in the IC interview. Across two questions, parents reported whether their child had an IC that was invisible and/or a stuffed animal with a distinct personality, and, if so, the frequency in which the child engaged in this type of play (1 = rarely, 5 = frequently). Children were also administered Singer and Singer’s (1981) imaginative play predisposition scale which assesses children’s favorite game, favorite toy, and whether they talk to themselves and what they think about prior to falling asleep. Those data have not been coded and are not included in the current analyses.

## Results

We first report the descriptive results of the anthropomorphism and role play measures, followed by analysis of the relation between anthropomorphism and role play. There were no gender differences for our dependent variables (*p*s > 0.14), thus subsequent analyses were collapsed across gender. Age differences were tested on all dependent variables and are reported where found.

### Anthropomorphism

Children’s scores on the IDAQ-CF were computed by averaging their responses across the eight technology-nature items (Technology-Nature subscale, α = 0.85), the four animal items (Animal subscale, α = 0.71), as well as across all 12 items (Overall scale, α = 0.79). Responses ranged from 0 (no endorsement of anthropomorphic beliefs) to 3 (full endorsement of anthropomorphic beliefs). Table 1 reports descriptive results by item and subscale for each age group. Children endorsed anthropomorphic beliefs about animals (*M* = 1.53, *SD* = 0.80) at a significantly higher rate than of technology and nature (*M* = 0.59, *SD* = 0.60), *t*(89) = -9.45, *p* < 0.001, *d* = 1.34. Significant age differences were found on the Animal subscale, *F*(2,87) = 4.43, *p* = 0.02. *Post hoc* analyses indicated that 5-year-olds (*M* = 1.26, *SD* = 0.79) endorsed lower levels of anthropomorphic beliefs about animals compared to 9-year-olds (*M* = 1.90, *SD* = 0.80), *p* = 0.01, *d* = 0.73 (see Table 1). Although 7-year-olds (*M* = 1.49, *SD* = 0.72) did not differ significantly from either 5- or 9-year-olds (*p*s > 0.18), those differences represented small to medium effects (*d* = 0.31 and *d* = 0.46, respectively) in the direction of increased anthropomorphism of animals with age. Although significant age differences were not found on the Technology-Nature subscale (*p* = 0.12), the direction trended towards reduced anthropomorphism of technology and inanimate nature with age. Age differences were not found on the Overall scale (*p* = 0.16). The differing developmental trajectories on the Animal subscale (significant increase with age) versus the Technology-Nature subscale (trending downward with age) underscore the important distinction between the subscales.

### Role Play

As described above, the role play scale included children’s and parents’ report of the child’s impersonation and whether they had an IC. Results are presented for each the impersonation scale and ICs.

#### Impersonation

Across three questions, children reported whether they impersonated animals, other people, and/or machines (i.e., pretending to be a cat/doctor/airplane). The vast majority of children (93.3%) reported impersonating at least one of these entities, and 91% of parents corroborated their child’s report. Results indicated that 78% of children reported pretending to be an animal, 62% pretended to be another person, and 42% pretended to be a machine. There were no age differences in any of the three forms of impersonation (*p*s > 0.34). We computed an Impersonation Score based on children’s report on the three questions (impersonation of an animal, another person, and/or machine), thus scores could range from 0 (no impersonation) to 3 (impersonation of all three types of entities). The mean Impersonation Score was 1.82 (*SD* = 0.87), with no age differences, *F*(2,87) = 0.58, *p* = 0.944.

#### Imaginary Companions (IC)

Sixty-five children (72.2 %) reported having an IC and substantiated their report with detailed descriptions of their IC during the interview (as described in the method). A 2 (IC type: toy or invisible) × 3 (age group) repeated-measures ANOVA revealed a main effect of IC type, indicating children were more likely to report having a toy IC (51.1%) than an invisible IC (21.1%), *F*(1,87) = 12.56, *p* < 0.001, η^2^= 0.13. Recall that this was a forced-choice question, thus children had to specify whether their IC was a toy or completely invisible. Largely consistent with children’s reports, 86% of parents corroborated their child’s report of an IC. There were no main effects of age group, *F*(2,87) = 0.22, *p* = 0.81, nor an interaction effect of IC type and age group, *F*(2,87) = 0.62, *p* = 0.54. The lack of significant age differences in IC type may be an artifact of our role play measure. That is, the IC rates include both current and former ICs. Recall that children were asked about their ICs (now or in the past), however, we did not ask them specify whether they were reporting on a current or former IC. Previous research (Taylor et al., 2004) has shown that *current* IC type differs by age with preschooler’s (3–5 years) being equally divided between toy and invisible ICs and 6- to 7-year-olds being twice and likely to have an invisible IC. Had we asked children to only report on current ICs, we may have seen age-related differences akin to Taylor et al. (2004). On the other hand, Pearson et al. (2001) found largely equal numbers of both current and former ICs between 5 and 9 years, and only after age 9 did children’s report of current ICs sharply decline. Thus, there are some discrepancies in the literature regarding the nature of age-related changes in IC type.

### Relationship Between Role Play and Anthropomorphism

Although we asked both children and parents about the child’s role play, we opted to use the child report, at times in conjunction with parent report where noted, in subsequent analyses of the relation between role play and anthropomorphism. This decision was guided by two reasons. First, as reported above, there was a high rate of parent corroboration (86% for ICs and 91% for impersonation) of child-reported role play. Second, as others have argued (e.g., Taylor et al., 2004), parent report alone is often incomplete as parents may not be fully aware of their children’s role play, especially with ICs in older children. Regarding the second point, we tested for age differences in parent-report of impersonation and ICs amongst those children who reported role play. Although we found no age differences in parent-report of impersonation (*p* = 0.42), we found marginally significant age differences in parent-report of ICs [*F*(2,61) = 2.97, *p* = 0.06] with 9-year-olds having the lowest rate of parent-report (76%). Thus, these results suggest that parents of 9-year-olds may be comparatively less informed about their children’s ICs.

We first examined the relation between impersonation and anthropomorphism. To do so, we used the computed Impersonation Scores, as a comprehensive measure of impersonation, to assess whether impersonation was predictive of anthropomorphism. Impersonation Scores were positively predictive of IDAQ-CF scores, after controlling for any effects of age, for both the overall scale (β = 0.28, *t* = 2.731, *p* = 0.008) and the Technology-Nature subscale (β = 0.24, *t* = 2.276, *p* = 0.025), but impersonation scores were only marginally predictive of the Animal subscale (β = 0.182, *t =* 1.809 *p* = 0.07, power = 0.801). In summary, overall impersonation of animals, people, and/or machines was positively related to the attribution of internal states to non-human entities, and in particular to inanimate nature and technology.

To further explore the relation between impersonation and anthropomorphism, we tested for differences between children who engaged or did not engage in the specific form of impersonation (animal or machine) on the corresponding form of anthropomorphism. No significant differences emerged. That is, children who impersonated animals were no different than those who did not in their anthropomorphism on the Animal subscale (*p* = 0.10, *d* = 0.42), nor did differences emerge on the overall scale (*p* = 0.07, *d* = 0.49) or Technology-Nature subscale (*p* = 0.25, *d* = 0.32). Similarly, those who impersonated machines did not differ from those who did not impersonate machines on the Technology-Nature subscale (*p* = 0.21, *d* = 0.27), nor did differences emerge on the overall scale (*p* = 0.25, *d* = 0.24) or Animal subscale (*p* = 0.81, *d* = 0.05). However, given that the effect sizes were very small to medium, these null results were likely a result of low power, as confirmed by a *post hoc* power analysis using G^∗^Power (Faul et al., 2007) indicating power ranged from 0.06 to 0.48. We also examined differences between children who impersonated people (*n* = 56) and those who did not (*n* = 34) and found significant differences in overall anthropomorphism (*p* = 0.05, *d* = 0.42), with impersonators anthropomorphizing more than non-impersonators. However, there were no significant differences between people impersonators and non-impersonators in animal anthropomorphism (*p* = 0.25, *d* = 0.25) or technology-nature anthropomorphism (*p* = 0.09, *d* = 0.38).

Given our prediction that higher levels of imagination should be related to a greater tendency to anthropomorphize, we then tested whether engagement in role play by role play type was differentially related to anthropomorphism. There is compelling evidence that the forms of role play are quite similar. Indeed, impersonation and ICs (toy and invisible) are both theorized to involve mental simulation (Harris, 2000) and both are related to theory of mind abilities albeit at different ages (Taylor and Carlson, 1997; Taylor et al., 2004). Yet, there is also evidence that role play types vary in important ways. For example, invisible ICs may procure additional benefits in imaginative abilities (e.g., Tahiroglu et al., 2011).

**FIGURE 1:**
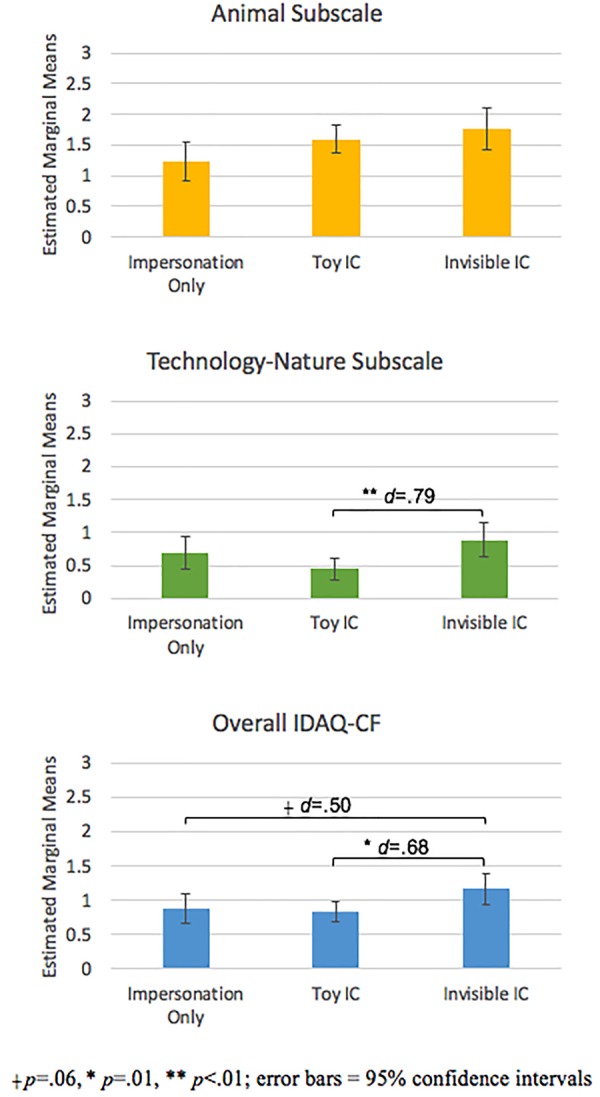
Mean IDAQ-CF scores by role play type.

As a first step, we classified children into one of three categories: impersonation only, toy IC, or invisible IC (Table 2). We then conducted one-way ANCOVAs to test for differences in anthropomorphism (analyzed using both the two subscales and the full scale) based on role play type while controlling for age. After controlling for potential age effects (*ns*), results indicated significant differences in role play type on the technology-nature subscale, *F*(2,83) = 4.215, *p* = 0.02, as well as the full scale, *F*(2,83) = 3.267, *p* = 0.04. As shown in Figure 1, subsequent pairwise comparisons indicated children with invisible ICs anthropomorphized technology and inanimate nature to a greater extent than those with toy ICs (*p* = 0.007), but did not differ from those children who only impersonated (*p* = 0.11). Similarly, on the overall scale, children with invisible ICs anthropomorphized more than children with toy ICs (*p* = 0.01), yet did not differ significantly from children who only impersonate (*p* = 0.06). Conversely, after controlling for significant age effects (*p* = 0.004), we did not find significant group differences on the animal subscale, *F*(2,83) = 1.449, *p* = 0.08, observed power = 0.51). However, the sample size was underpowered to detect the effects observed between children with toy or invisible ICs and those who only impersonated (*d* = 0.52 and *d* = 0.78, respectively).

**Table 2:** Proportions of role play type by age.

	Impersonation only	Imaginary companions	
		Toy	Invisible
5 years (*n* = 30)	0.30 (*n* = 9)	0.53 (*n* = 16)	0.17 (*n* = 5)
7 years (*n* = 29)	0.28 (*n* = 8)	0.45 (*n* = 13)	0.28 (*n* = 8)
9 years (*n* = 28)	0.18 (*n* = 5)	0.61 (*n* = 17)	0.21 (*n* = 6)
Total	0.25 (*n* = 22)	0.53 (*n* = 46)	0.22 (*n* = 19)

*Three children (one 7-year-old and two 9-year-olds) reported not engaging in any of these forms of role play, thus were excluded from these role play categories.*

Children also vary in the frequency with which they engage in the differing forms of role play, and is a marker of their engagement in role play. We were interested in examining direct effects of children’s frequency of engagement in role play on anthropomorphism, in addition to the interaction between frequency and role play type. To do so, we used standardized parent-reported frequency ratings (1 = rarely, 5 = frequently; *M* = 3.04, *SD* = 1.83) for each participant based on their role play category (impersonation only, toy IC, or invisible IC), as described above. Simple scatterplots suggested the relation may be curvilinear on the Technology-Nature subscale and overall scale, thus we examined both linear and curvilinear relations between role play frequency on both subscales and the overall scale as moderated by role play type (Dawson, 2014). To do so, we first dummy coded role play type using invisible ICs as the referent category to test for differences with toy ICs (Moderator 1 categorical variable) and impersonators (Moderator 2 categorical variable). We then ran a linear regression in Step 1 and curvilinear (quadratic) regression in Step 2 in order to test for model significance at each step (Table 3).

**Table 3:** Hierarchical linear and curvilinear (quadratic) regression analyses predicting anthropomorphism from frequency of engagement in role play and role play type.

Predictors	Model 1: Linear		Model 2: Quadratic	
	*b* [0.95 CI]	*p*	*b* [0.95 CI]	*p*
**Technology-Nature subscale**				
	Total *R*^2^= 0.14, Δ*R*^2^= 0.14, *p* = 0.06		**Total *R*^2^= 0.24, Δ*R*^2^= 0.10, *p* = 0.03**	
Constant	1.19 [0.59, 1.80]	<0.001	0.69 [0.001, 1.39]	0.05
Age (control)	–0.05 [–0.12, 0.03]	0.23	–0.05 [–0.13, 0.03]	0.20
Frequency	0.15 [–0.09, 0.38]	0.22	**0.36 [0.08, 0.64]**	**0.01**
Frequency^2^	–	–	**0.41 [0.07, 0.74]**	**0.02**
Moderator 1	**–0.43 [–0.75, –0.12]**	**0.008**	–0.05 [–0.61, 0.50]	0.86
Moderator 2	–0.17 [–0.52, 0.19]	0.36	0.54 [–0.06, 1.14]	0.08
Freq. × Mod. 1	–0.06 [–0.36, 0.23]	0.68	–0.20 [–0.55, 0.15]	0.26
Freq. × Mod. 2	–0.15 [–0.51, 0.22]	0.43	**–0.43 [–0.83, –0.03]**	**0.03**
Freq.^2^ × Mod. 1	–	–	–0.27 [–0.66, 0.12]	0.17
Freq.^2^ × Mod. 2	–	–	**–0.64 [–1.10, –0.18]**	**0.007**
**Animal subscale**				
	**Total *R*^2^= 0.17, Δ*R*^2^= 0.17, *p* = 0.03**		Total *R*^2^= 0.21, Δ*R*^2^= 0.07, *p* = 0.10	
Constant	0.67 [–0.16, 1.50]	0.11	0.45 [–0.51, 1.41]	0.36
Age (control)	**0.16 [0.05, 0.26]**	**0.005**	**0.14 [0.03, 0.24]**	**0.01**
Frequency	0.14 [–0.19, 0.46]	0.41	0.28 [–0.12, 0.67]	0.17
Frequency^2^	–	–	0.29 [–0.17, 0.75]	0.21
Moderator 1	–0.15 [–0.58, 0.28]	0.48	0.48 [–0.30, 1.25]	0.22
Moderator 2	**–0.52 [–1.01, –0.03]**	**0.04**	0.05 [–0.79, 0.89]	0.90
Freq. × Mod. 1	–0.10 [–0.51, 0.30]	0.61	–0.39 [–0.88, 0.09]	0.11
Freq. × Mod. 2	–0.13 [–0.63, 0.36]	0.59	–0.34 [–0.89, 0.22]	0.23
Freq.^2^ × Mod. 1	–	–	**–0.56 [–1.11, –0.02]**	**0.04**
Freq.^2^ × Mod. 2	–	–	–0.55 [–1.19, 0.10]	0.10
**Overall scale**				
	Total *R*^2^= 0.09, Δ*R*^2^= 0.09, *p* = 0.26		**Total *R*^2^= 0.19, Δ*R*^2^= 0.10, *p* = 0.04**	
Constant	1.01 [0.49, 1.54]	<0.001	0.62 [0.49, 1.54]	<0.001
Age (control)	0.02 [–0.05, 0.09]	0.57	0.01 [–0.06, 0.08]	0.74
Frequency	0.13 [–0.07, 0.34]	0.20	**0.32 [0.07, 0.56]**	**0.01**
Frequency^2^	–	–	**0.36 [0.07, 0.65]**	**0.02**
Moderator 1	**–0.33 [–0.60, –0.06]**	**0.02**	0.13 [–0.35, 0.61]	0.60
Moderator 2	–0.27 [–0.58, 0.04]	0.09	0.38 [–0.15, 0.90]	0.15
Freq. × Mod. 1	–0.07 [–0.32, 0.19]	0.60	–0.25 [–0.55, 0.05]	0.10
Freq. × Mod. 2	–0.13 [–0.44, 0.19]	0.42	**–0.38 [–0.73, –0.04]**	**0.03**
Freq.^2^ × Mod. 1	–	–	**–0.36 [–0.70, –0.02]**	**0.04**
Freq.^2^ × Mod. 2	–	–	**–0.60 [–1.00, –0.20]**	**0.004**

*The categorical moderator role play type was dummy coded with invisible IC as the referent, such that Moderator 1 = toy IC vs. invisible IC and Moderator 2 = impersonators vs. invisible IC. Bolded values indicate the best fitting model (linear or quadratic) for the subscales and overall scale, as well as significant predictors (*p* < 0.05) within each model.*

**FIGURE 2:**
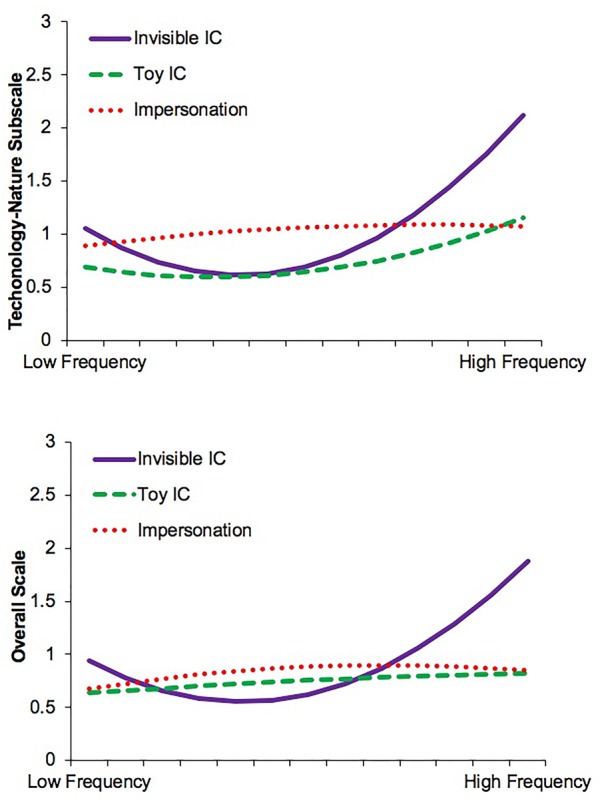
Moderating effect of role play type on the curvilinear relation between anthropomorphism and frequency of role play.

For the Technology-Nature subscale, the curvilinear (quadratic) model (Model 2) produced a significant increase in fit, *F*(6,78) = 3.28, *p* = 0.03, *R*^2^= 0.24 (Table 3). A significant curvilinear relation between role play frequency and anthropomorphism of technology and inanimate nature was found (Model 2: Frequency^2^, *p* = 0.02). This curvilinear relation was moderated by role play type when comparing children with invisible ICs to children who impersonated (Model 2: Freq.^2 ∗^ Mod. 2, *p* = 0.007), but not when compared to those with toy ICs (Model 2: Freq.^2 ∗^ Mod. 1, *p* = 0.17). As invisible ICs was the reference category, children with toy ICs and those who impersonated were not directly compared. Evidence of the curvilinear relation between role play frequency and anthropomorphism of technology and inanimate nature moderated by role play type is illustrated in Figure 2. We then tested whether a linear or curvilinear (quadratic) association was significant for each type of role play. For children with invisible ICs, the curvilinear relation between role play frequency and technology-nature anthropomorphism was significant, *b* = 0.12 [0.003, 0.23], *t*(2,15) = 2.20, *p* = 0.04, characterized by a concave (U-shaped) relation with a slight negative association when role play frequency was low and a stronger positive association as role play frequency increased from moderate to high. For children with toy ICs, the modest curvilinear relation was significant, *b* = 0.05 [0.003, 0.09], *t*(2,42) = 2.15, *p* = 0.04, characterized by a slightly positive association between frequency and technology-nature anthropomorphism at higher frequency of role play. Conversely, for children who impersonated, there was no significant linear or curvilinear relation (*p*s > 0.32) between role play frequency and technology-nature anthropomorphism.

For the animal subscale, the linear model produced a significant fit, *F*(6,78) = 2.57, *p* = 0.03, *R*^2^= 0.17, that was not significantly improved by the quadratic model (Table 3). After controlling for the significant effects of age, significant differences were found in animal anthropomorphism between children with invisible ICs and those who impersonated (Model 1: Moderator 2, *p* = 0.04), but not compared with children with toy ICs (Model 1: Moderator 1, *p* = 0.48). Role play frequency was not a significant predictor, nor was the interaction of frequency with role play type. Notably, the significant effect of role play type on anthropomorphism of animals diverges from the non-significant ANCOVA results. As noted above, the non-significant ANCOVA results may have resulted from insufficient power. A *post hoc* power analysis on the linear regression using G^∗^Power (Faul et al., 2007) with four predictors and an effect size of *f*^2^= 0.3 indicated observed power of 0.99, suggesting that the different statistical outcomes were due to power issues that were resolved in the regression.

Finally, on the overall scale, the curvilinear (quadratic) model produced the best fit, *F*(6,78) = 3.03, *p* = 0.04, *R*^2^= 0.19 (Table 3). There was a significant curvilinear relation between frequency of engagement and overall anthropomorphism (Model 2: Frequency^2^, *p* = 0.02). Role play type moderated that relation when comparing children with invisible ICs to those with toy ICs (Model 2: Freq.^2^ × Mod. 1, *p* = 0.04) and those who impersonated (Model 2: Freq.^2^ × Mod. 2, *p* = 0.004). Evidence of the curvilinear relation between role play frequency and overall anthropomorphism as moderated by role play type is illustrated in Figure 2. As with the technology-nature subscale, we then tested whether a linear or curvilinear (quadratic) association was significant for each type of role play. Children with invisible ICs had a significant U-shaped curvilinear relation, *b* = 0.11 [0.01, 0.21], *t*(2,15) = 2.26, *p* = 0.04, evidenced by a slight negative association between frequency of role play and overall anthropomorphism at low frequency of role play that became stronger and positive as frequency of role play increased from moderate to high. However, on the overall scale, there were no significant relations (linear or curvilinear) for children with toy ICs (*p*s > 0.65) or those who impersonated (*p*s > 0.21). Given that the overall scale disproportionately weights the eight technology and inanimate nature items relative to the four animal items, it is not surprising that the overall scale more closely resembles the Technology-Nature subscale than the Animal subscale.

## Discussion

### General Discussion

The present study provides initial evidence of a meaningful relation between children’s role play and anthropomorphism. This work marks a preliminary step toward addressing the question of whether role play and anthropomorphism are related, and potentially rely on a common simulation process of imagining others’ minds and internal states. Harris (1992, 2000) has argued that pretense (in the form of role play) involves a dual-process of simulating and projecting internal states, whether projecting others’ imagined mental states onto oneself (impersonation) or projecting imagined personalities onto a stuffed animal or invisible friend (ICs). We propose that anthropomorphism similarly involves the process of simulation and projection of internal states onto non-human others (e.g., animals, inanimate nature, or technologies). As an initial step in addressing this question, we reasoned that if role play and anthropomorphism involve a similar underlying process of simulation and projection of internal states and minds, then there should be a correspondence between children’s tendency to engage in role play and their tendency to anthropomorphize. Moreover, higher forms of role play should be related to attributing internal states to the least likely candidates (i.e., inanimate nature and technology), as both would involve greater imaginative processes.

The results from this study provide preliminary evidence consistent with our predictions. First, our findings indicate that the imaginative act of impersonation (i.e., pretending to be another entity) was positively related to anthropomorphism in general, and specifically the anthropomorphism of inanimate nature and technology. That is, children who impersonated more broadly across entities (animals, people, and/or machines) were more likely to anthropomorphize, especially inanimate nature and technologies. Whereas children who were more restrictive in who or what they impersonated tended to anthropomorphize less. Said differently, as this finding is correlational, children who more readily anthropomorphized were more likely to impersonate animals, people, and/or machines, and those who anthropomorphized less were less likely to impersonate.

Second, the results suggest that anthropomorphizing animals versus technology and inanimate nature may require differing *degrees* of imagination. Consider that animals provide numerous cues of agency and internal states (e.g., face, animate movement) – what has been termed ‘target triggers’ (Waytz et al., 2013) – and therefore may necessitate a lower level of imagination in order to attribute internal states. On the other hand, inanimate nature and technology lack such external cues of agency and internal states (i.e., target triggers), and therefore may require greater levels of imagination in order to attribute internal states. Given that, it is not surprising that children anthropomorphized animals more than technology and inanimate nature. Yet, over and above these differences, we found a pattern of results that suggest individual differences in role play may reflect differences in the degree to which children tap into the simulation process. That is, children who more readily imagine (or simulate) others’ mental states do so both in the context of role play and anthropomorphism.

The evidence in support of this suggestion comes in two forms. First, let us consider the differences found in anthropomorphism by role play type. The regression analysis indicated differences in anthropomorphism of animals by role play type: Although children with invisible ICs did not differ significantly from those with toy ICs, children with invisible ICs anthropomorphized animals significantly more than those who impersonated (recall that the ANCOVA was underpowered to statistically detect these group differences although the pattern was consistent with the regression analysis).

We also found differences by role play type in the anthropomorphism of technology and inanimate nature. Here, children with invisible ICs were more likely than those with toy ICs to anthropomorphize technology and inanimate nature. Arguably, children with invisible ICs are more advanced in their imaginative abilities, as an invisible IC lies completely within the realm of imagination. On the other hand, children with toy ICs may be constrained in their imaginative possibilities by the physical features of the toy, and as a result tap into the simulation process to a lesser degree. In line with this reasoning, Tahiroglu et al. (2011) found that 5-year-olds with invisible ICs demonstrated advanced imagery abilities compared to those with toy ICs. Moreover, children with invisible ICs have better theory of mind (Taylor and Carlson, 1997) and mental representation abilities (Taylor et al., 1993). Yet, we also found that children who exclusively impersonated (and did not have a toy or invisible IC) anthropomorphized technology and inanimate nature to nearly the same degree as children with invisible ICs. This finding appears counter to our expectation that more sophisticated forms of role play should be associated with greater tendencies to anthropomorphize. However, further consideration suggests this piece of evidence may be consistent with our premise. That is, children who exclusively engaged in impersonation are not bound in the personas they simulate. In any moment, they might pretend to be an astronaut, a rhinoceros, or a Martian. As a result, impersonators may be readily tapping into the simulation process as they impersonate a broad array of characters.

The second piece of evidence comes from our finding that children who most *frequently* engaged with their invisible ICs had the highest rates of anthropomorphizing technology and inanimate nature compared to all other role play groups. This finding suggests that the repetitive engagement in simulating and projecting mental states in one context may lead children to more readily engage in the simulation process in other contexts (role play to anthropomorphism, or vice versa). Intriguingly, the frequency of role play alone was not explanatory, rather the association between frequency and anthropomorphism (overall and technology-nature) depended upon the form of role play. Recall from the regression analysis that children with invisible ICs anthropomorphized technology and inanimate nature more as their frequency in engagement in this form of role play increased from moderate to high. However, children with toy ICs had only modest increases in anthropomorphism when their engagement in role play increased in frequency. Why might this be the case? Consider again our argument above that the form of role play may differentially draw upon the child’s imaginative abilities. Children with invisible ICs are completely unbound in the characters they create, and as a result may engage most substantively in the simulation process. As a result of greater imaginative abilities, in the absence of target triggers, these children may be better equipped or more inclined to ‘fill in the blanks’ with inanimate nature and technology. Thus, the level of imaginative ability associated with these more advanced forms of role play are associated with higher levels of mental state attribution to technology and inanimate nature. On the other hand, the imaginative potential for children with toy ICs might be limited by the physical features of the toy such that it is difficult for children to overcome the particular physical characteristics of a toy when imbuing it with a persona. As a result, they may engage less deeply in the simulation process – a limitation that is not wholly overcome by engaging more frequently in this form of role play.

Although children who impersonate have arguably fewer bounds in that they can impersonate any number of personas or characters, they nonetheless tend to impose limits by frequently impersonating familiar roles (e.g., mom) or storybook characters (e.g., Superman) (Carlson and Taylor, 2005), rather than generating a completely novel entity. This study did not assess the characters or persona children impersonated, thus we cannot know whether children who only engaged in impersonation tended to impersonate novel personas or known characters. However, our results do point to an interesting lack of relation between frequency of impersonation and anthropomorphism: Regardless of the frequency of impersonation, children who impersonated did not differ in their anthropomorphism (overall and technology-nature). The absence of this association stands in contrast to the significant curvilinear relation found for children with ICs – both showed increases in anthropomorphism of technology-nature and overall as the frequency of role play shifted from moderate to high. As with Carlson and Taylor (2005), it is possible that impersonators in the current study were often enacting known characters or roles rather than generating novel personas. In so doing, these children might be behaviorally enacting these characters, rather than deeply tapping into the simulation process of imagining these character’s internal states.

Why might it be the case that higher levels of imagination are associated with more anthropomorphism? We argue above that by more deeply engaging in the simulation process, children may be more inclined or equipped to simulate and project mental states in other contexts. Here, we lay out an additional, complimentary explanation that helps unpack why children may anthropomorphize the least likely candidates for mental state attribution: inanimate nature and technology. Carlson (2010) has argued that higher levels of imagination involve a freeing up of top-down conscious control in order to allow for greater imaginative products. Accordingly, it may be that children with greater imaginations (vis-à-vis having a frequent invisible IC) may use less conscious control, in general, when imagining others’ minds and internal states and are able to more readily attribute internal states to the least obvious candidates – inanimate nature and technology. In other words, these children are not constrained by the lack of target triggers or external cues of mental states when they simulate and project mental states, whether onto an invisible friend or technology and inanimate nature.

Finally, in light of the positive relation between role play and anthropomorphism, one lingering question regards children’s level of commitment to their anthropomorphic beliefs. To explore this question, we must first unpack a critical difference between role play and anthropomorphism. In the case of role play, children are quite clear on the distinction between pretense and reality (Woolley and Wellman, 1993; Taylor, 1999; Gleason, 2013; Woolley and Ghossainy, 2013). That is, although children may be immersed within the imaginary space, they maintain a clear grasp on what is real and what is pretend. Accordingly, in terms of level of commitment, children have low commitments to their pretend attributions (i.e., they know they are just pretend). Conversely, individuals may express varying levels of commitment to their attributions when anthropomorphizing, in line with Epley et al.’s (2008) weak and strong forms of anthropomorphism. Weak anthropomorphism reflects a low-level of commitment, wherein individuals engage in ‘in-the-moment’ mindless (non-deliberate) behaviors that are not substantiated by their explicit judgments (e.g., Nass et al., 1993; Nass and Moon, 2000; Kim and Sundar, 2012). For example, one may act as if their computer has intentions (e.g., “You always try to update right when I need to give a presentation!”), and at the same time not explicitly believe their computer has intentions. Thus, like pretense, weak anthropomorphism involves a divergence between a person’s explicit claims and their in-the-moment behaviors. However, the difference between pretense and weak anthropomorphism lies in the individual’s level of awareness: One is aware of their pretense, but more often likely to be mindless when engaging in weak anthropomorphism (Nass and Moon, 2000). Strong anthropomorphism, on the other hand, is marked by explicit commitment to anthropomorphic beliefs, and a consistency between one’s behaviors and their expressed beliefs (e.g., believing their dog has emotions and treating them accordingly).

What form of anthropomorphism – weak or strong – do children’s attributions reflect? Given that our study measured anthropomorphism with an explicit measure (IDAQ-CF), we argue that our results reflect the strong form of anthropomorphism. That is, we argue that explicit anthropomorphic attributions are more likely to reflect a higher level of commitment. At the same time, children were judicious in the degree to which they attributed anthropomorphic characteristics to non-humans. Recall that children’s attributions were nowhere near ceiling levels, but rather were at the lower-end (for inanimate nature and technology) and mid-point (for animals) of the 3-point scale. Thus, children may be committed to their attributions, even when they are conservative in the degree to which they endorse anthropomorphic attributes (e.g., being sure that an insect thinks for itself only a little).

### Limitations

The current study has several limitations that warrant consideration. First, this study involved a single-time-point correlational design to investigate the relation between role play and anthropomorphism. Although the results are consistent with our proposal that role play and anthropomorphism involve a common process of simulation and projection, any firm conclusion to that effect would be premature and go beyond the existing data or study design. Certainly, questions about causal relationships between role play and anthropomorphism would require time-lagged, longitudinal, or experimental designs. Second, our anthropomorphism measure relied exclusively upon self-report. Although we found variability in children’s use of the IDAQ-CF scale (thus rendering unlikely a yes bias in their responses), other factors may have affected how children responded on this measure. Our role play scale similarly relied upon children’s self-report, however, in this case their reports were largely corroborated by their parent. That said, additional behavioral measures of role play, such as free play with real- and fantasy-oriented toys, pretend actions (Taylor and Carlson, 1997), or the toy phone task (Taylor et al., 1993; Tahiroglu et al., 2011), would provide a more comprehensive and robust measure of engagement in role play. Third, the two measures were presented in the same order (IDAQ-CF followed by the Role Play Measure), thus we were unable to assess or control for potential order effects. Finally, this work should be replicated to guard against the possibility of a spurious finding and would be strengthened by including a larger (i.e., to increase power) and more representative sample.

### Future Directions

Research on the development of anthropomorphism is in its nascent stage, and much work remains in order to understand the causes, correlates, and consequences of the tendency to ascribe human-like mental states to animals, artifacts, and nature. Indeed, the current study is a starting point for understanding the relation between role play and anthropomorphism. Future work here should focus on investigating the specific mechanisms that may underlie a general process of mind attribution. For example, do more general cognitive abilities explain the relation between role play and anthropomorphism? Accordingly, future research would benefit from the addition of measures of cognitive abilities (e.g., executive function, theory of mind, analogical reasoning), as well as other control measures associated with mentalizing abilities (e.g., birth order, multilingualism). It will also be important to test whether anthropomorphism can be causally linked to role play, for example by experimentally testing whether increases in role play would result in increases in anthropomorphism. Importantly, future work examining frequency of engagement in role play needs to be considered in light of the form of role play, as the curvilinear relation between frequency of engagement and anthropomorphism of technology and nature and in general is moderated by role play type. Future research could also assess whether the nature of the relation between role play type and anthropomorphism undergoes a qualitative shift across age groups. In addition, the current study has raised questions regarding children’s level of commitment to their anthropomorphic attributions. We have argued above that children’s explicit judgments on the IDAQ-CF reflect a strong form of anthropomorphism (i.e., high commitment), however, this interpretation is conceptual rather than empirical. Thus, future research could directly assess how committed children are to their attributions, and whether their level of commitment undergoes developmental change. That is, a strong commitment to anthropomorphic beliefs may reflect a less advanced understanding. Whereas, a lower level of commitment to one’s anthropomorphic attributions may reflect a more sophisticated and nuanced appreciation that ascertaining whether non-human others have mental states is a challenging, if not futile, task (e.g., Nagel, 1974).

In addition, we suggest three distinct lines of research that are particularly relevant to understanding the development of anthropomorphism, as well as the variation between individuals (whether innate or a result of experience) and cultures.

We have argued that, like role play, anthropomorphism involves a process for ascribing mental states onto others, whether a toy or stuffed animal (in the case of role play) or a non-human entity (in the case of anthropomorphism). Relatedly, Harris (2000) has argued that simulation underlies both role play and theory of mind. Previous research has shown links between pretense (and role play, specifically) and theory of mind (e.g., Astington and Jenkins, 1995; Taylor and Carlson, 1997). Although, as pointed out by Dore et al. (2015), there is conflicting evidence regarding any directionality between pretense and theory of mind. One interpretation of the conflicting directional evidence is a third variable: Both may involve a common underlying process. The current paper makes a third link—that is, the speculation that simulation also underlies anthropomorphism. In other words, it is possible that the process of simulation and projection of internal states to others includes other people (theory of mind), imagined others (role play), and non-human others (anthropomorphism).

Thus, one line of future research might explore the relation between anthropomorphism and theory of mind (see also Atherton and Cross, 2018). As previously discussed, there is evidence that anthropomorphism activates the same neural network as theory of mind (Castelli et al., 2000, 2002). Interestingly, Castelli et al. (2002) found that, in response to viewing anthropomorphized animated shapes, individuals with high-functioning autism provided fewer and less accurate interpretations of putative mental states and showed less activation of the mentalizing network. At the same time, individuals with autism demonstrated similar activation as typical adults of an additional region – the extra-striate visual cortex. However, unlike typical adults, those with autism had poor connectivity between the extra-striate cortex region and the mentalizing network. The authors suggest the results point to a physiological explanation for theory of mind deficits among individuals with autism; that is, information from lower-level perceptual (visual processing) areas is not transmitted to the higher-level mentalizing network. These results provide neural evidence of a link between theory of mind and anthropomorphism in typical adult, as well as a neural explanation for the difficulty individuals with autism have interpreting animate shapes in mental terms.

Yet, there may be a critical difference between *perceiving* animated objects in mentalistic terms and explicitly *ascribing* them mental states. A recent study found that roughly half of adults with autism spontaneously personify objects (White and Remington, 2018). This finding may call into question the logic of our argument that social cognition and anthropomorphism are related. White and Remington suggest this result is particularly striking given that roughly half of autistic individuals experience difficulties identifying their own emotion (alexithymia). However, as participants’ emotion understanding – their own or others’ emotions – were not assessed (nor other aspects of theory of mind), it is not possible to know whether the 56% of participants with autism who reported personifying objects also experienced alexithymia. Nevertheless, these results underscore the importance of additional research on any links between attributing mental states to humans (theory of mind) and non-humans (anthropomorphism). It may be, as White and Remington suggest, that anthropomorphism “may result from difficulties mentalizing” (p. 3). To this point, we propose two related avenues of inquiry. First, it will be important to explore potential links between anthropomorphism and *accuracy* in theory of mind understanding (e.g., emotion understanding, perspective taking, knowledge attribution, etc.). This line of investigation would shed light on whether the tendency to anthropomorphize is associated with a *lack* of accuracy in understanding other people’s minds, or vice versa. Second, and relatedly, future research should also consider the relation between anthropomorphism and the *propensity* to make inferences about others’ minds, what has been termed mind-reading motivation (Carpenter et al., 2016). Carpenter et al. (2016) have found that one’s accuracy in interpreting others’ mental states is distinct from (although weakly related to) their propensity or motivation to do so. Thus, independent of individual’s accuracy in mind-reading, future work could investigate whether anthropomorphism is more likely to arise in individuals with a greater willingness to attribute mental states to others – what we may think of as ‘promiscuous social cognition.’

Finally, a critical question regarding any potential link between theory of mind and anthropomorphism is whether the association would be the same for animals as it is for technology and inanimate nature. As evident in the current study and previous research (Waytz et al., 2010a; Severson and Lemm, 2016), these forms of anthropomorphism are distinct. As we argued above, animals may be more readily anthropomorphized as they provide numerous external cues of internal states. Accordingly, the simulation process applied to humans (theory of mind) may be more akin to that applied to animals as both provide external cues that may more readily allow for inferences of internal states. However, technology and inanimate nature provide few, if any, external cues and therefore may draw upon different aspects of theory of mind and/or other cognitive abilities (e.g., visual imagery). Thus, the distinction between anthropomorphism of animals and anthropomorphism of technology and inanimate nature should be maintained in future work.

A second line of future research might investigate whether anthropocentric biases may interact with children’s tendency to anthropomorphize. Anthropocentrism refers to the tendency to use humans as a prototype for reasoning inductively about non-humans, wherein children asymmetrically extend unobservable novel biological properties from a human to a target animal, plant, or object, but not vice versa (Carey, 1985). Interestingly, rather than being a foundation for inferring knowledge about non-humans as initially theorized (Carey, 1985), subsequent research has shown that experience and social learning play an important role in children’s anthropocentric biases (Waxman and Medin, 2007; Herrmann et al., 2010). Compared to urban children, children from rural environments and Native American communities do not exhibit an anthropocentric bias, presumably due to more direct experience with animals and nature (Bang et al., 2007; Medin et al., 2010). Moreover, children in urban environments show less of an anthropocentric bias when they have pets (Inagaki, 1990; Geerdts et al., 2015) or parents with biological expertise (Tarlowski, 2006). Although anthropocentrism and anthropomorphism are arguably distinct (e.g., anthropocentrism is evident among urban children for a relatively brief period, whereas anthropomorphism is found in children and adults; Geerdts, 2016), future research could explore whether anthropocentrism is related to anthropomorphism. On the surface, it stands to reason that they would be associated to the extent that they both involve the attribution of unobservable internal characteristics (whether biological or mental) from humans to non-humans. Indeed, there is evidence that anthropomorphic storybooks can influence children’s tendency to reason anthropocentrically (Waxman et al., 2014). Alternatively, it may be that substantive differences exist between anthropocentrism and anthropomorphism, especially when considering differences in culture and experience, in addition to differing underlying cognitive processes involved in conceptual understanding versus social cognition.

Finally, a third promising line of research would explore cultural variation in anthropomorphism. As discussed above, it seems likely that culture would play a role in anthropomorphic beliefs as metaphysical beliefs and societal norms differ widely. Some have also argued that cultural differences in self-construal (i.e., perceptions of self as independent versus part of a collective) may procure differences in the tendency to perceive minds in non-humans (Waytz et al., 2013). In the adult literature, there is evidence of universality in agency detection in human faces (Looser and Wheatley, 2010) and inferences of intentions based on motion (Barrett et al., 2005), yet there is a surprising lack of cross-cultural research on mind attribution to non-humans. Anthropological study has provided initial evidence of cultural differences; that is, primatologists in Japan anthropomorphize to a greater extent than their United States counterparts (Asquith, 1986). In a more recent study with adults, Ghuman et al. (2015) found higher rates of anthropomorphism (as measured by the IDAQ; Waytz et al., 2010a) among adults in China and India compared to United States adults. Therefore, this line of research is ripe with opportunity to identify the patterns and causes of cultural variation in anthropomorphic beliefs.

## Conclusion

The present study provided initial evidence of a link between children’s role play and anthropomorphism. We proposed that role play and anthropomorphism involve a common simulation process of mental state attribution, and our results were consistent with this proposal insofar that a positive relation was found between the tendency to engage in role play and the tendency to anthropomorphize. Moreover, our results provide evidence that differing degrees of imagination are involved in anthropomorphism of animals versus technology and inanimate nature. Future work is needed to corroborate the link found in the current study and, importantly, to identify whether there are specific underlying mechanisms. More generally, research on the cognitive underpinnings of anthropomorphism is in its beginning stages, and it represents an area rich with interesting and important questions.

## Data Availability Statement

The raw data supporting the conclusions of this manuscript will be made available by the authors, without undue reservation, to any qualified researcher.

## Author Contributions

Both authors made substantial intellectual contributions to this paper and approved it for publication. RS conceived the study, collected the data, analyzed the data, and wrote the manuscript. SW analyzed the data and wrote the manuscript.

## Conflict of Interest Statement

The authors declare that the research was conducted in the absence of any commercial or financial relationships that could be construed as a potential conflict of interest.
